# Laparoscopic vs. open mesorectal excision for rectal cancer: Are these approaches still comparable? A systematic review and meta-analysis

**DOI:** 10.1371/journal.pone.0235887

**Published:** 2020-07-28

**Authors:** Maria Conticchio, Vincenzo Papagni, Margherita Notarnicola, Antonella Delvecchio, Umberto Riccelli, Michele Ammendola, Giuseppe Currò, Patrick Pessaux, Nicola Silvestris, Riccardo Memeo

**Affiliations:** 1 General Surgery, Policlinico di Bari, Bari, Italy; 2 Department of Health Sciences, General Surgery, Magna Græcia University, Medicine School of Germaneto, Catanzaro, Italy; 3 IRCAD-IHU, General, Digestive, and Endocrine Surgery, University of Strasbourg, Strasbourg, France; 4 Medical Oncology Unit, IRCCS Cancer Institute "Giovanni Paolo II", Bari, Italy; 5 Department of Biomedical Sciences and Human Oncology, University of Bari ‘Aldo Moro’, Bari, Italy; 6 Department of Hepato-Pancreato-Biliary (HPB) Surgery, Miulli Hospital, Acquaviva delle Fonti, Bari, Italy; Sapienza University of Rome, ITALY

## Abstract

**Background:**

To analyze pathologic and perioperative outcomes of laparoscopic vs. open resections for rectal cancer performed over the last 10 years.

**Methods:**

A systematic literature search of the following databases was conducted: Cochrane Central Register of Controlled Trials, MEDLINE (through PubMed), EMBASE, and Scopus. Only articles published in English from January 1, 2008 to December 31, 2018 (i.e. the last 10 years), which met inclusion criteria were considered. The review only included articles which compared Laparoscopic rectal resection (LRR) and Open Rectal Resection (ORR) for rectal cancer and reported at least one of the outcomes of interest. The analyses followed the Preferred Reporting Items for Systematic Reviews and Meta-analysis (PRISMA) statement checklist. Only prospective randomized studies were considered. The body of evidence emerging from this study was evaluated using the Grading of Recommendations Assessment Development and Evaluation (GRADE) system. Outcome measures (mean and median values, standard deviations, and interquartile ranges) were extracted for each surgical treatment. Pooled estimates of the mean differences were calculated using random effects models to consider potential inter-study heterogeneity and to adopt a more conservative approach. The pooled effect was considered significant if p <0.05.

**Results:**

Five clinical trials were found eligible for the analyses. A positive involvement of CRM was found in 49 LRRs (8.5%) out of 574 patients and in 30 ORRs out of 557 patients (5.4%) RR was 1.55 (95% CI, 0.99–2.41; p = 0.05) with no heterogeneity (I^2^ = 0%). Incorrect mesorectal excision was observed in 56 out of 507 (11%) patients who underwent LRR and in 41 (8.4%) out of 484 patients who underwent ORR; RR was 1.30 (95% CI, 0.89–1.91; p = 0.18) with no heterogeneity (I^2^ = 0%). Regarding other pathologic outcomes, no significant difference between LRR and ORR was observed in the number of lymph nodes harvested or concerning the distance to the distal margin. As expected, a significant difference was found in the operating time for ORR with a mean difference of 41.99 (95% CI, 24.18, 59.81; p <0.00001; heterogeneity: I^2^ = 25%). However, no difference was found for blood loss. Additionally, no significant differences were found in postoperative outcomes such as postoperative hospital stay and postoperative complications. The overall quality of the evidence was rated as high.

**Conclusion:**

Despite the spread of laparoscopy with dedicated surgeons and the development of even more precise surgical tools and technologies, the pathological results of laparoscopic surgery are still comparable to those of open ones. Additionally, concerning the pathological data (and particularly CRM), open surgery guarantees better results as compared to laparoscopic surgery. These results must be a starting point for future evaluations which consider the association between ‘‘successful resection” and long-term oncologic outcomes. The introduction of other minimally invasive techniques for rectal cancer surgery, such as robotic resection or transanal TME (taTME), has revealed new scenarios and made open and even laparoscopic surgery obsolete.

## 1. Introduction

Colorectal cancer is the third most common type of malignant tumors worldwide [1]. There were over 1.8 million new cases in 2018 [2]. Rectal cancer accounts for approximately one third of all colorectal cancers [3], presenting higher local recurrence rates and reduced disease-free survival [4] as compared to colonic tumors. In addition, rectal cancer had an increased risk of postoperative complications and long-term morbidity. Considering the complexity of the management of this kind of cancer, it is essential to rely on a multidisciplinary team in order to guarantee the assessment of the best treatment path.

The ideal treatment for rectal cancer remains surgical resection, and an important step has been taken towards the creation of training programs for specialist surgeons. A number of studies have demonstrated the impact of a dedicated team on oncologic outcomes, complication rates, and long-term clinical outcomes in patients with rectal cancer [5].

Total Mesorectal Excision (TME) represents a milestone in rectal cancer surgery, introduced as an improvement to the already existing surgical techniques according to RJ Heald [6]. Prior techniques such as Miles’ resection or anterior resection were however burdened by major recurrence rates [7]. With Heald’s revolution, local recurrence rates at 5 years were reduced from 30–40% [8] to 3.7% [9].

In addition, the analysis of the mesorectum also provided key information on the quality of surgery based on macroscopic and microscopic specimens [10].

In recent times, minimally invasive surgery was increasingly used in oncologic surgery, due to its benefits on postoperative outcomes (earlier recovery and return to bowel function, shorter length of hospital stay) as confirmed in previous meta-analyses [11]. Additionally, laparoscopic surgery for rectal cancer has gained popularity thanks to the development of technical skills, associated with increasing surgeons’ expertise.

Although a routine use of laparoscopy is still discussed and studied, recent multicentric studies, namely the COLOR II trial [12] and the COREAN trial [13], compared the laparoscopic with the open approach in rectal cancer resection, showing that laparoscopic resection was associated with more favorable short-term outcomes with no significant differences in terms of oncologic results as compared to open resection. In other randomized trials, ACOSOG Z6051 and ALaCaRT evaluated composite benchmarks of an adequate surgical resection, such as completeness of total mesorectal excision and negativity of circumferential and distal margins (CRM, DRM) associated with short- and long-term outcomes. With similar results, these last two studies reported that the non-inferiority of laparoscopic surgery compared to open surgery for rectal cancer was not established [14, 15].

The introduction of transanal Total Mesorectal Excision (taTME) [16] stands for an increasingly less invasive approach.

Considering recent publications [17, 18], we conducted a systematic review and meta-analysis of randomized clinical trials (RCTs) comparing laparoscopic rectal resection (LRR) versus open rectal resection (ORR).

The aim of our work was to evaluate pathologic outcomes and short-term clinical outcomes of laparoscopic surgery as compared to open surgery in patients with rectal cancer. Our review only enrolled articles published over the last ten years in order to understand if the widespread use of laparoscopic surgery had increased pathologic outcomes and short-term results in rectal cancer.

## 2. Materials and methods

### 2.1. Study design and inclusion criteria

This systematic review and meta-analysis considered prospective studies which compared laparoscopic rectal resection (LRR) with open rectal resection (ORR) for rectal cancer and which reported at least one of the outcomes of interest. The analyses followed the Preferred Reporting Items for Systematic Reviews and Meta-analysis (PRISMA) statement checklist [19]. Eligibility criteria were established before initiating the data research to ensure an appropriate selection.

Only prospective randomized studies were considered, and no trial duration limitation was applied. All included studies compared LRR with ORR and were included irrespective of the surgical technique and the performance of a primary anastomosis. Participants only included adult patients with a histologically proven rectal cancer requiring rectal resection.

All studies were reviewed (methods and analyses) in compliance with ethical principles for medical research.

The following primary pathologic outcomes were considered: circumferential resection margin (CRM) involvement (distance from the tumor to the closest cut edge of the tissue ≤1mm), nonomplete mesorectal excision (defined in the classification established by Nagtegaal et al. [20]), number of lymph nodes harvested, and the distance between the tumor and the distal margin.

Secondary clinical outcomes included operating time, blood loss, postoperative hospital stay, and postoperative complication rates.

Study selection criteria were defined according to the following PICO framework:

Population: adult patients with rectal cancer
Intervention: rectal resection
Comparison: laparoscopic approach versus open approach

No robotic or transanal minimally invasive approach was performed.

Outcomes included pathologic outcomes (CRM involvement, complete TME, number of harvested lymph nodes, distance between tumor and distal resection margin). Clinical outcomes included operating time, blood loss, postoperative hospital stay, and postoperative complication rates.

### 2.2. Exclusion criteria

Patients with tumor other than a histologically proven adenocarcinoma and people younger than 18 years were excluded.

Other exclusion criteria were as follows: body mass index (BMI calculated as weight in kilograms divided by the square of height in meters) greater than 30; ECOG(Eastern Cooperative Oncology Group) performance score of 3 or more (range: 3–5, with higher scores indicating a higher disability); patients not receiving neoadjuvant CRT or RT; operation not performed within 4 to 12 weeks after final radiation treatment; history of invasive pelvic malignancy within 5 years; psychiatric or addictive disorders which affected adherence to the protocol; ASA (American Society of Anesthesiologists) classification IV or V; [10] severe systemic disease; conditions that limited the success of laparoscopic resection; and life expectancy of less than 12 weeks.

Additionally, our review excluded tumors which involved circumferential resection margin pretreatment, T1 tumor treated with local transanal excision, T3 rectal cancer within 2mm from the endopelvic fascia; tumors larger than 6cm; patients with a history of other malignant neoplasms except basal-cell carcinoma or in situ carcinoma of the cervix uteri.

Other exclusion criteria were as follows: patients with acute abdomen or who underwent emergency surgery for acute intestinal obstruction or tumor perforation or patients requiring synchronous colorectal surgery; familial adenomatous polyposis coli/hereditary non–polyposis colorectal cancer; active inflammatory bowel disease such as Crohn’s disease or ulcerative colitis; pregnancy was also excluded, neoadjuvant CRT; distant metastasis; previous abdominal operations near the region of the colorectal operation; recurrent disease; ongoing infection or plasma neutrophil level of less than 2 × 109/L; associated gastrointestinal tract disease necessitating surgical intervention; transanal local excision or transcoccygeal excision; bulky tumor larger than 8cm in diameter unsuitable for epidural insertion.

### 2.3. Literature search and selection

A combination of the following terms was adopted for literature search: cancer/carcinoma/rectal/colorectal/surgery/therapy/treatment/management/laparoscopy/laparoscopic/laparotomy/trial/randomized trial. The search was performed in the following databases: Cochrane Central Register of Controlled Trial, MEDLINE (through PubMed), EMBASE, and Scopus. To identify additional relevant studies, the reference list of the eligible studies and review articles were checked. Only articles published in English from January 1, 2008 to December 31, 2018 (namely over the last 10 years) which met inclusion criteria were considered.

According to the CONSORT 2010 Statement for RCTs (http://www.consort-statement.org), two reviewers (MC and VP) screened the titles and abstracts of the retrieved studies for relevance purposes. The screening of each reviewer was independent and blind. Articles excluded by both reviewers were removed. In a second step, the reviewers performed full-text analyses of the selected articles and independently assessed the risk for bias using the Cochrane tool for assessing risk for bias [21].

In addition, the body of evidence emerging from this study was evaluated using the Grading of Recommendations Assessment Development and Evaluation (GRADE) system [22]. All disagreements between the two reviewers during the selection/evaluation processes were resolved via discussion with a third reviewer (RM).

### 2.4. Statistical analyses

Data from the included articles were processed using qualitative and quantitative analyses. For binary outcome data, the relative risk (RR) and 95% CI were estimated using the Mantel-Haenszel method. For continuous data, the mean differences (MD) and 95% CIs were estimated using inverse variance weighting. Outcome measures (mean and median values, standard deviations, interquartile ranges) were extracted for each surgical treatment. If the SE was provided instead of an SD, the SD was calculated on the basis of sample size (SE = SD/). When neither mean nor SD values were reported, they were estimated from the median ranges, interquartile ranges (IQR) or p values [23]. Heterogeneity was assessed using the I2 statistic [21, 24]. I2 values of 25, 50, and 75% were considered low, moderate, and high respectively [21, 24].

Pooled estimates of the mean differences were calculated using random effects models to consider potential inter-study heterogeneity and to adopt a more conservative approach. The robustness of the results and the potential sources of heterogeneity were then explored by performing sensitivity analyses (e.g., subgroup analyses; comparison using a fixed effects model). The pooled effect was considered significant if p <0.05. The meta-analysis was performed using Review Manager (RevMan, version 5.3, Cochrane Collaboration, Copenhagen, Denmark).

## 3. Results

**Fig 1:**
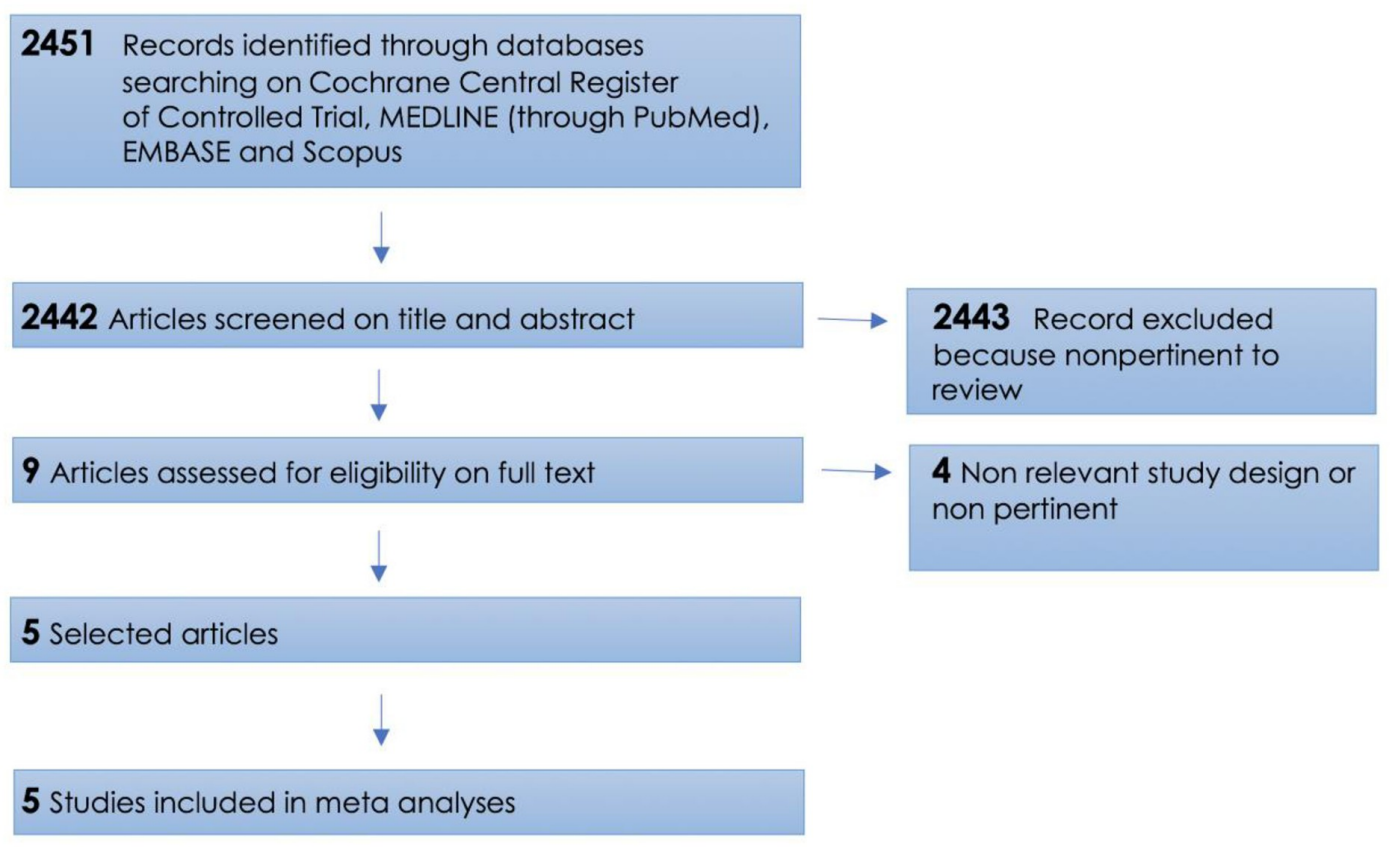
A PRISMA flow diagram of the inclusion of studies.

Our search identified 2,451 articles, and 2,442 of them were rejected on title and abstract selection. After removal of duplicates, nine publications were fully reviewed. Five clinical trials were found eligible for the meta-analyses. A PRISMA flow diagram showing the process of inclusion of studies is displayed in Fig 1.

The 5 selected studies were all published from 2012 to 2015. All randomized controlled studies (RCS) included patients who underwent surgery during the last 10 years. Overall, a total of 1,216 patients undergoing rectal surgery for cancer were analyzed (unique patients).

The groups included 638 and 578 patients for LRR and ORR respectively (Table 1).

**Table 1:** Summary of included randomized clinical trials.

SOURCE	N° OF INSTITUTIONS (COUNTRY) AND STUDY PERIOD	CRITERIA		TOT N° OF PARTECIPANTS (N° INCLUDED)	N° PROCEDURES (N° INCLUDED)		AGE, MEAN(SD), y		TYPES OF PROCEDURES(%)		NEOADJUVANT THERAPY		CONVERSION TO ORR (%)
		INCLUSION	EXCLUSION		LRR GROUP	ORR GROUP	LRR GROUP	ORR GROUP	LRR GROUP	ORR GROUP	LRR GROUP	ORR GROUP	
**Fleshman et al, 2015**	35, USA and Canada Oct2008-sept2013	Cancer≤12 cm from AV	1–11	462	240	222	57,7 (11,5)	57,2 (12,1)	AR 179(74.6)APR 58(24.2)H 1(0.4);TPC 2(0.8)	AR 169(76.1);APR 47(21.2);TPC6(2.7)	242 (100)	239 (100)	27/240 (11.2)
**Stevenson et al, 2015**	24, Australia and New Zeland Mar2010-Nov2014	T1-T3; cancer ≤15 cm from AV	1;2;4;7;10;12;13	473	238	235	65 (5.2)	64.75 (4.9)	AR 207(89);APR 18(11);	AR 210(90); APR 15(10)	119 (50)	116 (48.9)	22/238 (8.8)
**Fujii et al, 2013**	Japan, Aug2008-Aug 2012	Age ≥75y; istological ADH; stage ≥ T4a	26;17;15;21;29;41;11;28	58	29	29	79,8 (3,6)	80,1 (4,2)	HAR 5(5);LAR 19(19);APR4(4);ISR 1(1)	HAR 7(7);LAR 19(19);APR 2(2);H 3(3);			
**Gong et al, 2012**	Shangai cancer center, Sept2008-Jul2011	Cancer ≤10cm from AV;stageII-III	11;13;26	138	67	71	58,4 (13,6)	59,6 (9,4)					2/67 (0.02)
**Kennedy, 2014**	12,UK Jul2008-Apr2012	age≥18y;any stage	11;42;29	56	29	27	69,3 (9,4)	70,1 (8,7)	AR 22(76.9);APR 5(17.2);OTH2(6.9)	AR 20(74.1);APR 6(22.2);OTH1(3.7)	14(a) (13.6)	10(a) (9.9)	

a pre operative radiotherapy

**Exclusion criteria are defined as follows**:(1)other tumor than histologically proven adenocarcinoma; (2)younger than 18 years; (3)body mass index (BMI; calculated as weight in kilograms divided by height in meters squared) greater than 34; (4)Eastern Cooperative Oncology Group performance score of 3 or more (range, 3–5, with higher scores indicating higher disability); (5)not receiving neoadjuvant CRT or RT; (6)operation not performed within 4 to 12 weeks of the final radiation treatment; (7)history of invasive pelvic malignancy within 5 years; (8)psychiatric or addictive disorders that affected adherence to the protocol; (9)American Society of Anesthesiologists classification IV or V; (10)severe systemic disease; (11)conditions that limit the success of laparoscopic resection; (12)life expectancy of less than 12 weeks; (13)T4 tumors or involved circumferential resection margin pretreatment; (14T1 tumor treated with local transanal excision; (15history of other malignant neoplasm except basocellular carcinoma of the skin or in situ carcinoma of the cervix uteri; (16) signs of acute intestinal obstruction; (17)need for synchronous colorectal surgery; (18)familial adenomatous polyposis coli/hereditary nonpolyposis; (19)colorectal cancer; (20)active Crohn disease or ulcerative colitis; (21)pregnancy; (22) T3 rectal cancer within 2 mm from the endopelvic fascia; (23)tumor perforation; (24)tumor larger than 6 cm; (25)neoadjuvant CRT; (26)distant metastasis; (27)distal tumor needing anastomosis within 5 cm of the dentate line; (28)previous abdominal operations near the region of the colorectal operation; (29) emergency surgery; (30) recurrent disease; (31) ongoing infection or plasma neutrophil level of less than 2 × 109/L; (32) associated gastrointestinal tract disease needing surgical intervention; (33) malignant disease in the past 5 years; (34) BMI greater than 30; and (35) previous abdominal surgery; (36) APR; (37) multiple cancer; (38) distance of tumor from anal verge≥5cm; (39) multiple colorectal cancer; (40) transanal local excision or transcoccygeal excision; (41) bulky tumor llarger than 8 cm in dm; (42) unsuitable for epidural insertion.

Different types of surgical procedures were performed (AR for anterior resection, APR for abdominoperineal amputation, H for Hartmann’s procedure, TPC for total proctocolectomy, LAR for low anterior resection, ISR for intersphincteric resection).

Among the 638 laparoscopic operations, 52 required conversion to ORR.

### 3.1. Study quality assessment

Considering the Cochrane Collaboration’s tool for assessing risk of bias in randomized trials [21], the five studies selected for the meta-analysis were classified as having a low risk of bias [25–29], and the quality of the evidence was rated as high according to the GRADE system [22].

### 3.2 Pathologic and clinical outcomes

**Fig 2:**
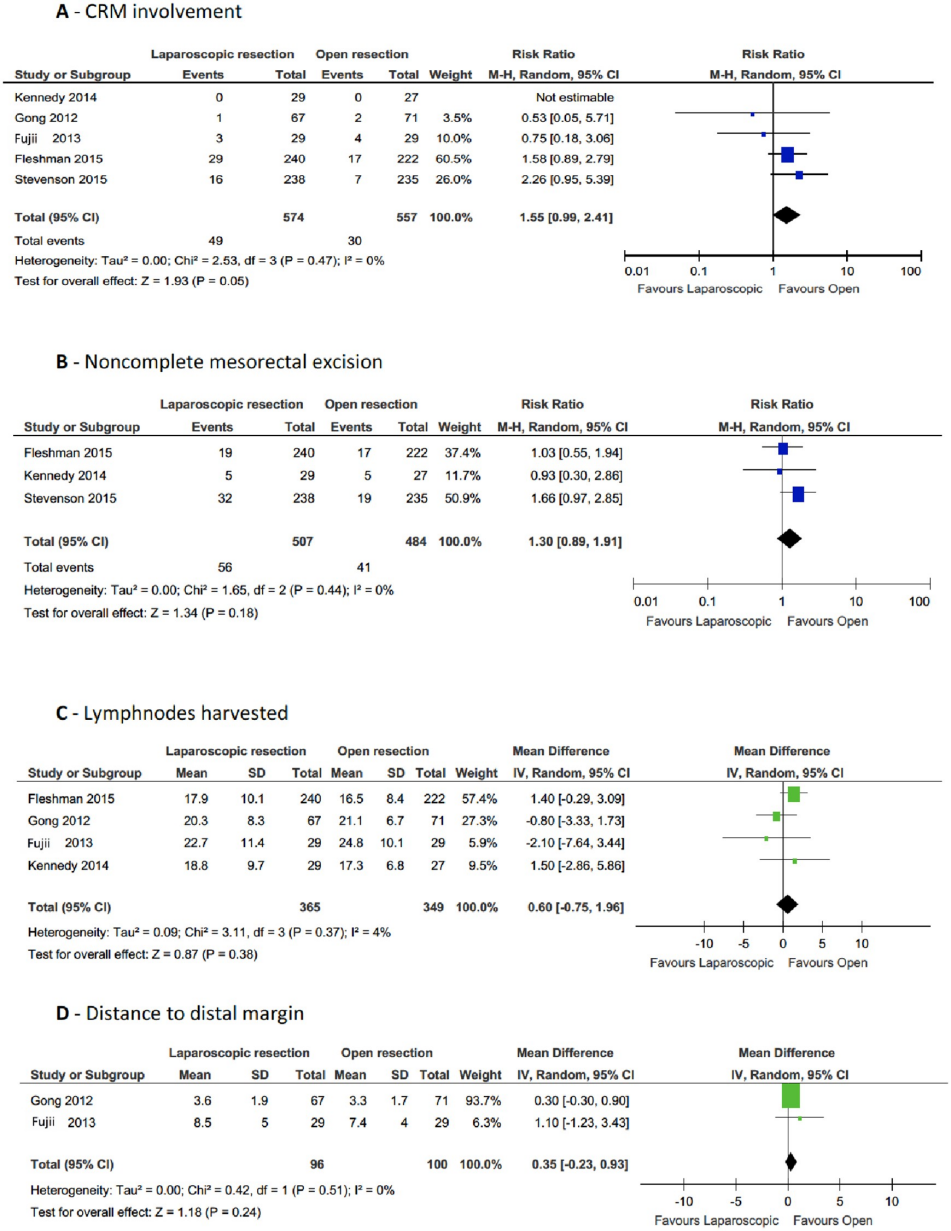
Forest plot of pathological outcomes.

Four out of five studies reported CRM involvement. CRM was considered positive when ≤1mm. Data from Kennedy et al. [27] were not included because they considered the shortest distance from the tumor and CRM as pathologic outcome. From the four eligible studies, a positive CRM involvement was found in 49 LRR of 574 patients and in 30 ORR of 557 patients; RR for CRM involvement was 1.55 times higher in LRRs than in ORRs (95% CI, 0.99–2.41; p = 0.05) with good heterogeneity (I^2^ = 0%) (Fig 2A).

Three RCTs reported the rate of non-complete mesorectal excision. The cumulative risk of sub-optimal mesorectal excision was 1.3 higher for LRRs than for ORRs, even if this result was not statistically significant (95% CI, 0.89–1.91; p = 0.18, I^2^ = 0%) (Fig 2B).

With respect to other pathologic outcomes, no significant difference between LRR and ORR was observed in the number of lymph nodes harvested or concerning the distance to the distal margin.

Four studies reported the mean number of lymph nodes harvested; the mean difference was 0.60, slightly in favor of ORR (95% CI, -0.75, 1.96; p = 0.38; heterogeneity: I^2^ = 4%) (Fig 2C). Two RCTs reported the distance to the distal margin with a mean difference of 0.35 between LRRs and ORRs, in favor of ORR (95% CI, -0.23, 0.93; p = 0.24; no heterogeneity: I^2^ = 0%).

Concerning clinical outcomes, we compared operating time, intraoperative blood loss quantification, postoperative hospital stay, and postoperative complication rates.

**Fig 3:**
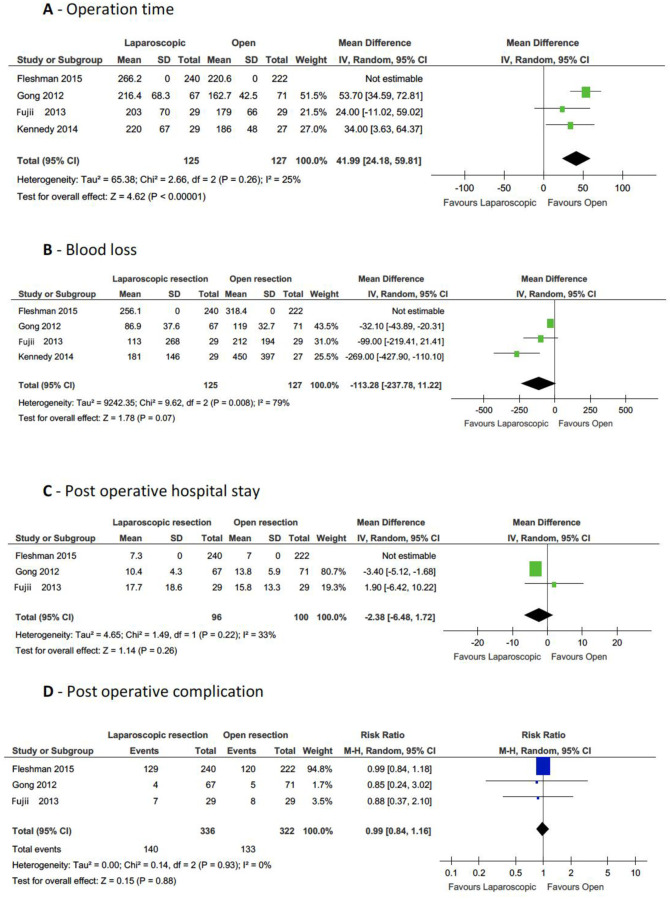
Forest plot of peri-operative and clinical outcomes.

Operating time was reported by three studies. As expected, a significant difference was found in the operating time in favor of ORR with a mean difference of 41.99 (95% CI, 24.18, 59.81; p <0.00001; heterogeneity: I^2^ = 25%) (Fig 3A).

The difference in blood loss between LRR and ORR was not statistically significant. However, it was markedly in favor of LRR, even if there was a moderate heterogeneity (mean difference -113.28; 95% CI, -237.78, 11.22; p = 0.07; I2 = 79%) (Fig 3B). Additionally, no significant differences were found in postoperative outcomes such as postoperative hospital stay or postoperative complications (Fig 3C–3D).

Postoperative hospital stay was reported in two RCTs. The mean difference was -2.38 days shorter if LRR had been performed (95% CI, -6.48, 1.72; p = 0.26; heterogeneity: I^2^ = 33%).

The postoperative complication rate was provided in three studies. A relative risk of 0.99 was assessed, excluding differences between the LRR and the ORR approach, with optimal heterogeneity (I^2^ = 0%, 95% CI, 0.84–1.16; p = 0.88).

## 4. Discussion

This systematic review and meta-analysis focused on the pathologic outcomes of laparoscopic resection (LRR) versu open resection (ORR) for rectal cancer, as highlighted by RCT data only, over the last 10 years.

Our study underlined comparable pathologic outcomes for LRR and ORR regarding mesorectal complete excision, number of lymph nodes harvested, and distal margin distance. Nonetheless, CRM involvement is better demonstrated in open surgery. In addition, in the evaluation of short-term outcomes, there were no significant differences between LRR and ORR.

One of the most debated and major fields of comparison was the evaluation of total mesorectal excision (TME) quality and CRM involvement. Since its introduction by RJ Heald et al. [9], the concept of TME has been defined as the gold standard in rectal cancer surgery. The crucial point for the execution of mesorectum excision lies in its initial phase, and particularly so in the recognition of the “holy plane”.

Not only does the integrity of the resected mesorectum represent an essential prognostic factor [30], but it also has significant repercussions on circumferential resection margins. The importance of CRM involvement was already described by Quirke in 1986, and numerous subsequent studies have confirmed its prognostic value in local recurrence.

In recent times, we have witnessed a major revolution in terms of surgical techniques, due to the introduction of minimally invasive surgery which has rapidly spread in the field of oncologic surgery. The introduction of laparoscopy in colorectal surgery has brought many advantages such as the refinement of surgical technique on one side, and clinical advantages for patient on the other side.

From a surgical standpoint, the laparoscopic approach offers an ideal gain. Indeed, the magnified view allows for a better exposure of appropriate planes in the narrow pelvic space. As a result, genitourinary disfunction [31] and blood loss can be more easily prevented. However, the laparoscopic approach still remains technically challenging, as described in the literature, for patients with a high BMI, for advanced tumor stages, lower tumors, and after neoadjuvant therapy [32].

The most important advantage of laparoscopic surgery was represented by a faster recovery than in open surgery, as reported in the COLOR II trial [12]. This study highlighted that the clinical gain of laparoscopy remains evident only in the short postoperative period. However, as far as longer functional results are concerned, no difference was shown in nerve-sparing strategies [33].

Our study seemed to be in agreement with results of recent meta-analyses [34, 35], which stated that open surgery guarantees better pathologic results despite the impressive technical progress in minimally invasive rectal surgery, and despite minimal improvements in oncologic results.

Our meta-analysis showed a significantly higher risk of positive CRM involvement in LRRs as compared to ORRs (8.5 vs. 5.4% respectively, p = 0.05).

Other proofs in the literature led to the same conclusion. Indeed, the ACOSOG Z6051 reported a negative CRM involvement in 87.9% of laparoscopic resections and in 92.3% of open resections. The ALaCaRT study indicated positive circumferential resection margins in 7% of the laparoscopic group as compared to 3% of the open groups [14, 15].

The cut-off value most frequently used in the literature to define a positive CRM is >1mm. However, this is still under discussion. Nevertheless, some authors suggested that 2mm or less rather than 1mm or less defined a positive CRM. This is a possible source of disarray and increased heterogeneity in the literature [36].

Considering that the best oncologic result for the patient is the objective of surgery, and even in rectal surgery, the number of harvested nodes, complete TME, and the distance from the distal margin represent essential parameters in order to evaluate the completeness of surgical resection.

The results achieved from the evaluation of CRM involvement are confirmed by other variables examined in our study with a special focus on non-complete mesorectal excision reported in three RCTs, which play a keyrole as a prognostic value for local recurrence and overall survival. Non-complete mesorectal excision was observed in 11% of patients who underwent LRR and in 8.4% of patients who underwent ORR, in line with the results of a recent meta-analysis by De Angelis et al. [35]. It was demonstrated that the rate of non-complete total mesorectal excision was significantly higher in the laparoscopic group while no significant differences were highlighted regarding CRM and DRM involvement when compared with the open surgery group.

Additionally, no significant difference between LRR and ORR were observed in the number of lymph nodes harvested with a mean difference of 0.60 in 4 out of 5 studies, or in the distance to the distal margin with a mean difference of 0.30 considering only 2 out of 5 investigations.

In two of the trials considered (ACOSOG Z6501 and ALaCaRT), ‘‘successful resection” was described as a composition of variables (negative CRM, complete or near-complete TME, and negative DRM), which could well provide a more specific “surgical report” and would represent a prognostic factor for pathologic outcomes.

The impact of laparoscopic surgery on blood loss, operating time, length of hospital stay, and postoperative complication rates was evaluated considering the perioperative course. As expected, a significant difference was found in the operating time in favor of ORR, reported in three studies, with a mean difference of 41.99 (p <0.00001). The learning curve for laparoscopic resection may account for this finding. However, according to Zhang’s study, the operating time and the rate of accumulation are inversely proportional to the number of procedures and time respectively [37].

Regarding blood loss, postoperative hospital stay, and postoperative complications, there were no statistically significant difference between the two groups (LRR and ORR), although higher rates in favor of laparoscopic surgery were observed. Improvement in surgical skills make it possible to better recognize the planes and the correct ligation of vessels allow for reduced bleeding, in association with the use of appropriate tools for coagulation.

This type of data is not highlighted in our study since our analysis is more focused on comparing pathologic outcomes as opposed to clinical outcomes in laparoscopic surgery versus open surgery. In spite of this, as described in the literature, minimally invasive approaches allow for a more rapid recovery, a shorter hospital stay, and according to some studies, for a lower percentage of morbidity and short-term mortality [38].

The search for an increasingly minimally invasive technique is constantly becoming weaker and seems to find excellent answers in robotic and transanal surgery, although for none of these very two approaches has a superiority been already established as compared to the laparoscopic approach.

Robotic surgery in rectal cancer is a valid attempt to respond to the limits of laparoscopy due to reduced physiologic tremor, allowing a tridimensional vision and an expanded range of movements, and this has a noticeable impact on improving the visualization of surgical planes and subsequently contributes to an accurate resection, especially in male obese patients and/or patients with tumors of the middle and low rectum. Initial data from a multicenter ROLARR study showed no difference in the positivity rate of pathologic circumferential resection margins (CRM) [39].

Merely patients with neoplasia of the middle and lower rectum represented an indication for the transanal approach according to Lacy et al. Not only does this type of approach seem to guarantee a better visualization of the distal resection margin, and therefore improved pathologic outcomes, but it also seems to offer an increased guarantee of nerve-sparing and sphincter-saving surgery, with major repercussions on clinical outcomes. These data originate from the COLOR III study, which although it is still in its recruitment phase, seem to indicate superiority of taTME compared to laparoscopy in terms of oncologic outcomes [40] according to the investigators’ expectations. There is growing interest in the transanal approach for colorectal surgeons, although it presents a considerable complexity in terms of surgical technique. For this reason, the need for systematic and standardized training programs would contribute to the correct acquisition of surgical skills and expertise [41].

Considering the even more frequent approach of young surgeons to minimally invasive surgery, the possibility of achieving better surgical skills in less time, reducing the duration of the learning curve, obtaining similar pathologic results compared to open surgery holds great promise for the future. The introduction of new technologies, as for instance intraoperative navigation and augmented reality, will surely represent an essential support to improve oncologic results in surgical patients.

### 4.1. Limitations

This meta-analysis takes into account randomized controlled trials only, as they are considered the best level of statistical evidence. In spite of this, no bias can be completely excluded. For instance, the chemotherapeutic schemes are not superimposable in the various investigations included. Likewise, the adopted surgical techniques (anterior resections, abdominoperineal amputations, and Hartmann’s resection) result in different percentages in the RCTs. Considering only the last 10 years, a limitation is represented by the difference of proven standardization for open and laparoscopic techniques unlike new robotic and transanal approaches. Additionally, the exclusion of some ultralow rectal tumor resection can change our statistical analysis of results. However, beyond such limitations, the low degree of heterogeneity and sensitivity of the analysis effectively supports the consistency and value of our data.

## 5. Conclusions

Our review and meta-analysis demonstrated that the oncologic results of laparoscopic surgery were still comparable to those of open surgery through the dissemination of laparoscopy with dedicated surgeons and the development of even more sophisticated surgical tools and technologies. In addition, data on short-term clinical outcomes were not significant as expected. These results must be a starting point for future evaluations, which consider the association between ‘‘successful resection” and long-term oncologic outcomes in RCTs to select one of the two approaches.

## Supporting information

S1 Data (PDF)

S1 Checklist PRISMA 2009 checklist. (PDF)
