# Supplementary material for: Laparoscopic vs. open mesorectal excision for rectal cancer: Are these approaches still comparable? A systematic review and meta-analysis
Source: PLoS One. 2020 Jul 28;15(7):e0235887. doi: 10.1371/journal.pone.0235887 (PMC7386630; doi:10.1371/journal.pone.0235887)
Supplement: S1 Data — (PDF) [file pone.0235887.s001.pdf]

	Number of patients		CRM involvement [n(%)]	Non complete mesorectal excision [n(%)]	Lymph nodes harvested [mean(SD)]	DRM, cm [mean(SD)]	Operation time, min [mean, (SD)]	Blood loss, ml [mean(SD)]	Post-operative hospital stay, days [mean(SD)]	Post-operative complications n patients (%)
**Gong et al, 2012**	LRR	67	1 (1.5)	-	20.3 (8.3)	3.6 (1.9)	216.4 (68.3)	86.9 (37.6)	10.4 (4.3)	4 (5.9)
	ORR	71	2 (2.8)	-	21.1 (6.7)	3.3 (1.7)	162.7 (42.5)	119 (32.7)	13.8 (5.9)	5 (7)
**Ishibe et al, 2013**	LRR	29	3 (10.3)	-	22.7 (11.4)	8.5 (5.0)	203 (70)	113 (268)	17.7 (18.6)	7 (24.1)
	ORR	29	4 (13.8)	-	24.8 (10.1)	7.4 (4.0)	179 (66)	212 (194)	15.8 (13.3)	8 (27.6)
**Kennedy et al, 2014**	LRR	29	-	5 (17.2)	18.8 (9.7)	-	220 (67)	181 (146)	-	-
	ORR	27	-	5 (18.5)	17.3 (6.8)	-	186 (48)	450 (397)	-	-
**Fleshman et al, 2015**	LRR	240	29 (12.1)	19 (7.9)	17.9 (10.1)	-	266.2 *	256.1*	7.3*	129 (53.8)
	ORR	222	17 (7.6)	11 (4.9)	16.5 (8.4)	-	220.6*	318.4*	7 *	120 (54)
**Stevenson et al, 2015**	LRR	238	13 (5.5)	32 (13.4)	-	-	-	-	-	-
	ORR	235	7 (3)	19 (12.3)	-	-	-	-	-	-

*SD not reported in the source article

CRM circumferential resection margin, DRM distal resection margin
